# CANT1 deficiency in a mouse model of Desbuquois dysplasia impairs glycosaminoglycan synthesis and chondrocyte differentiation in growth plate cartilage

**DOI:** 10.1002/2211-5463.12859

**Published:** 2020-04-23

**Authors:** Kazuki Kodama, Hiroaki Takahashi, Nobuyasu Oiji, Kenta Nakano, Tadashi Okamura, Kimie Niimi, Eiki Takahashi, Long Guo, Shiro Ikegawa, Tatsuya Furuichi

**Affiliations:** ^1^ Laboratory of Laboratory Animal Science and Medicine Co‐Department of Veterinary Medicine Faculty of Agriculture Iwate University Morioka Japan; ^2^ Department of Laboratory Animal Medicine Research Institute National Center for Global Health and Medicine (NCGM) Tokyo Japan; ^3^ Laboratory of Laboratory Animal Science and Medicine School of Veterinary Medicine Kitasato University Towada Japan; ^4^ Section of Animal Models Department of Infectious Diseases Research Institute National Center for Global Health and Medicine (NCGM) Tokyo Japan; ^5^ Support Unit for Animal Resources Development, Research Resources Division RIKEN Center for Brain Science Saitama Japan; ^6^ Laboratory for Bone and Joint Diseases RIKEN Center for Integrative Medical Sciences Tokyo Japan; ^7^ Department of Basic Veterinary Science United Graduate School of Veterinary Science Gifu University Japan

**Keywords:** CANT1, chondrocyte, Desbuquois dysplasia, extracellular matrix, genome editing, glycosaminoglycan

## Abstract

Desbuquois dysplasia (DD) type 1 is a rare skeletal dysplasia characterized by a short stature, round face, progressive scoliosis, and joint laxity. The causative gene has been identified as calcium‐activated nucleotidase 1 (*CANT1*), which encodes a nucleotidase that preferentially hydrolyzes UDP to UMP and phosphate. In this study, we generated *Cant1* KO mice using CRISPR/Cas9‐mediated genome editing. All F0 mice possessing frameshift deletions at both *Cant1* alleles exhibited a dwarf phenotype. Germline transmission of the edited allele was confirmed in an F0 heterozygous mouse, and KO mice were generated by crossing of the heterozygous breeding pairs. *Cant1* KO mice exhibited skeletal defects, including short stature, thoracic kyphosis, and delta phalanx, all of which are observed in DD type 1 patients. The glycosaminoglycan (GAG) content and extracellular matrix space were reduced in the growth plate cartilage of mutants, and proliferating chondrocytes lost their typical flat shape and became round. Chondrocyte differentiation, especially terminal differentiation to hypertrophic chondrocytes, was impaired in *Cant1* KO mice. These findings indicate that CANT1 is involved in the synthesis of GAG and regulation of chondrocyte differentiation in the cartilage and contribute to a better understanding of the pathogenesis of DD type 1.

## Graphical abstract

Mutations in the gene encoding for CANT1 (calcium‐activated nucleotidase 1) are responsible for Desbuquois dysplasia (DD) type 1. *Cant1 *KO mice exhibit skeletal defects that recapitulate DD type 1. Reduced glycosaminoglycan (GAG) content and impaired chondrocyte differentiation are observed in the growth plate cartilage of these mutants. These findings indicate that CANT1 is involved in the synthesis of GAG and regulation of chondrocyte differentiation.

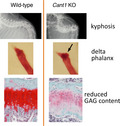

**Abbreviations**
DD: Desbuquois dysplasia
*CANT1*: calcium‐activated nucleotidase 1
GAG: glycosaminoglycan
CS: chondroitin sulfate
DS: dermatan sulfate
HS: heparan sulfate
KO: knockout
WT: wild‐type
ECM: extracellular matrix
IHH: Indian hedgehog

## Introduction

Desbuquois dysplasia (DD) is a rare skeletal dysplasia inherited as an autosomal recessive trait and is characterized by a short limb short stature, progressive scoliosis, and round face and joint laxity [1, 2]. DD is clinically heterogeneous and classified into two types based on the presence (type 1) or absence (type 2) of characteristic hand anomalies. These anomalies consist of an extra ossification center distal to the second metacarpal, delta phalanx, bifid distal thumb phalanx, and dislocation of the interphalangeal joints [3, 4]. An additional variant of DD has been proposed, called the DD Kim variant, which is characterized by advanced carpal bone age and shortness of one or all metacarpal bones, with elongated appearance of the phalanges but no accessory ossification center [5]. The causative gene for DD type 1 and Kim variant has been identified as *CANT1*, which encodes calcium‐activated nucleotidase 1 [6, 7, 8], and the causative gene for DD type 2 has been identified as *XYLT1*, which encodes xylosyltransferase 1 [9, 10]. In the formation of proteoglycans, a family of extracellular macromolecules comprised of glycosaminoglycan (GAG) chains linked to a central core protein [11, 12], xylosyltransferase 1 functions as the initiator of GAG chain biosynthesis by transferring xylose from UDP xylose to the core protein [13, 14, 15]. Meanwhile, CANT1 is a nucleotidase that preferentially hydrolyzes UDP to UMP and phosphate [16, 17, 18]. CANT1 has also been predicted to be involved in GAG synthesis, and the recent study using genetically modified mice has confirmed the prediction [6, 7, 8, 19].

Glycosaminoglycans are large linear polysaccharides constructed of repeating disaccharides and can be classified into chondroitin sulfate (CS), dermatan sulfate (DS), heparan sulfate (HS), and heparin, based on their disaccharide units. GAG chains are modified by sulfation at various hydroxyl group positions and also by the epimerization of uronic acid residues [13, 14, 15]. This structural diversity plays important roles in a range of biological functions including development, growth, and homeostasis of skeletal tissues. In humans and animals, many skeletal dysplasias are caused by mutations in genes encoding glycosyltransferases, sulfotransferases, and related enzymes responsible for the biosynthesis of GAGs [14, 15]

Paganini and colleagues generated two mouse strains by an ES cell‐based method: a *Cant1* knockout (KO) and a *Cant1* knock‐in harboring DD type 1 causative p.R302H missense mutation [19]. Both strains present skeletal dysplasia phenotypes similar to DD type 1. Moreover, biochemical studies have demonstrated that CANT1 deficiency causes abnormal GAG synthesis in the cartilages, including reduced GAG content and length, and GAG oversulfation. This indicates that CANT1 is critical for GAG biosynthesis in the cartilage. However, because the expression of chondrocyte‐specific marker genes in these mutants has not been examined, the effects of CANT1 deficiency on chondrocyte differentiation have remained unclear. Further, histology of growth plate cartilage in the previous models was only examined until 3 weeks of age, with unknown continuation. In this study, we generated a novel *Cant1* KO mouse strain using CRISPR/Cas9‐mediated genome editing and addressed these additional issues.

## Materials and methods

### Mice and ethical statement

Mice were housed in a temperature‐controlled room with a 12‐h/12‐h light/dark cycle and fed with standard mouse laboratory chow with free access water. They were sacrificed with an overdose of pentobarbital or by decapitation. All animal experiments were approved by the Animal Experimentation Committee at Iwate University (Approval No. A201810) and the National Center for Global Health and Medicine (Approval No. 18037).

### Genome editing

CRISPR/Cas9‐mediated genome editing in mice was performed as described previously, with some modifications [20]. Briefly, crRNA for the target sequence (5’‐ATTCGGTACCGAATCCCACC‐3’) and tracrRNA were synthesized by Fasmac Co., Ltd. (Kanagawa, Japan), and recombinant Cas9 protein (EnGen Cas9 NLS) was purchased from New England Biolabs Inc. (Ipswich, MA, USA). The crRNA (0.15 pmol·μL^−1^), tracrRNA (0.15 pmol·μL^−1^), and Cas9 protein (22.5 ng·μL^−1^) were co‐injected into the cytoplasm of fertilized eggs derived from C57BL/6J mice (Japan SLC, Hamamatsu, Japan). After the injected oocytes were cultured overnight *in vitro*, two‐cell embryos were transferred into pseudo‐pregnant female mice. Genomic DNA was isolated from tail samples of the offspring using standard methods. The edited region around exon 3 of the *Cant1* gene was amplified by PCR, using the following primers: 5′‐GCCTCAGACTAAATGTTGTTCCAAGT‐3′ and 5′‐GAAATGGCGGACCAGCTGTTCTGA‐3′. The amplification products were sequenced, and their sequences were compared to the reference sequence.

### X‐ray examination and skeletal preparation

Radiographs were obtained using a TRS‐1005 soft X‐ray apparatus (Saffron, Tokyo, Japan). Sacrificed mice were eviscerated and fixed in 99% EtOH for 4 days. Alcian blue staining was performed in a solution of 80% EtOH, 20% acetic acid, and 0.015% Alcian blue for 4 days at 37 °C. Specimens were then rinsed and soaked in 95% EtOH for 3 days. Alizarin red staining was then performed in a solution of 0.002% Alizarin red and 1% KOH for 12 h at room temperature. After rinsing with water, specimens were kept in 1% KOH solution until the skeletons became clearly visible. For storage, specimens were sequentially transferred into 50%, 80%, and finally 100% glycerol.

### Histological analysis

Limbs dissected from sacrificed mice were fixed in 4% paraformaldehyde, decalcified in 10% EDTA for 1 week at 4 °C, and embedded in paraffin. Hematoxylin and eosin and Safranin O staining were performed using 6‐μm paraffin sections according to standard protocols.

### Western blot analysis

Tissue pieces were homogenized in chilled RIPA butter with proteinase inhibitors. Proteins (20 μg per lane) were separated using SDS/PAGE gels and transferred to PVDF membranes. The membranes were incubated in 5% BSA in TBS‐T to block nonspecific binding. Membranes were incubated with a CANT1 primary antibody (C‐3, Santa Cruz Biotechnology, Dallas, TX, USA) at 1 : 1000 dilution with Can Get Signal Immunoreaction Enhancer Solution 1 (TOYOBO, Tokyo, Japan) and then with goat anti‐mouse IgG‐HRR (sc‐2005, Santa Cruz Biotechnology) at 1: 10 000 dilution with Can Get Signal Solution 2. The bands were visualized with Clarity Western ECL Substrate (Bio‐Rad, Hercules, CA, USA).

### *In situ* hybridization analysis

Mouse limbs were fixed with G‐Fix (Genostaff, Tokyo, Japan) at room temperature and decalcified with G‐Chelate Mild (Genostaff). The decalcified samples were then embedded in paraffin on CT‐Pro20 (Genostaff) using G‐Nox (Genostaff) and sectioned at 5 μm. Digoxigenin‐labeled RNA probes were synthesized by *in vitro* transcription using DIG RNA Labeling Mix (Roche Diagnostics, Mannheim, Germany). Hybridization was conducted using an ISH Reagent Kit (Genostaff). Tissue sections were deparaffinized with G‐Nox and rehydrated using an ethanol series and PBS. The sections were fixed with 10% formalin in PBS for 30 min at 37 °C, placed in 0.2% HCI for 10 min at 37 °C, and then treated with 3 μg·mL^−1^ of Proteinase K (Wako Pure Chemicals, Osaka, Japan) in PBS for 10 min at 37 °C. Sections were then re‐fixed with 10% formalin in PBS for 10 min at room temperature and placed in 0.2 N HCl for 10 min at room temperature. Hybridization was performed with RNA probes at concentrations of 250 ng·mL^−1^ in G‐Hybo‐L (Genostaff) for 16 h at 60 °C. Staining was performed with an NBT/BCIP solution (Sigma‐Aldrich) overnight. Sections were counterstained with methyl green.

### Statistical analyses

Student’s *t*‐tests were used to determine the significance of differences between wild‐type (WT) and *Cant1* KO mice or control (WT + heterozygous *Cant1* KO) and *Cant1* KO mice groups. Significance was defined as *P* < 0.05.

## Results

### Generation of *Cant1* KO mice by genome editing

**Fig. 1:**
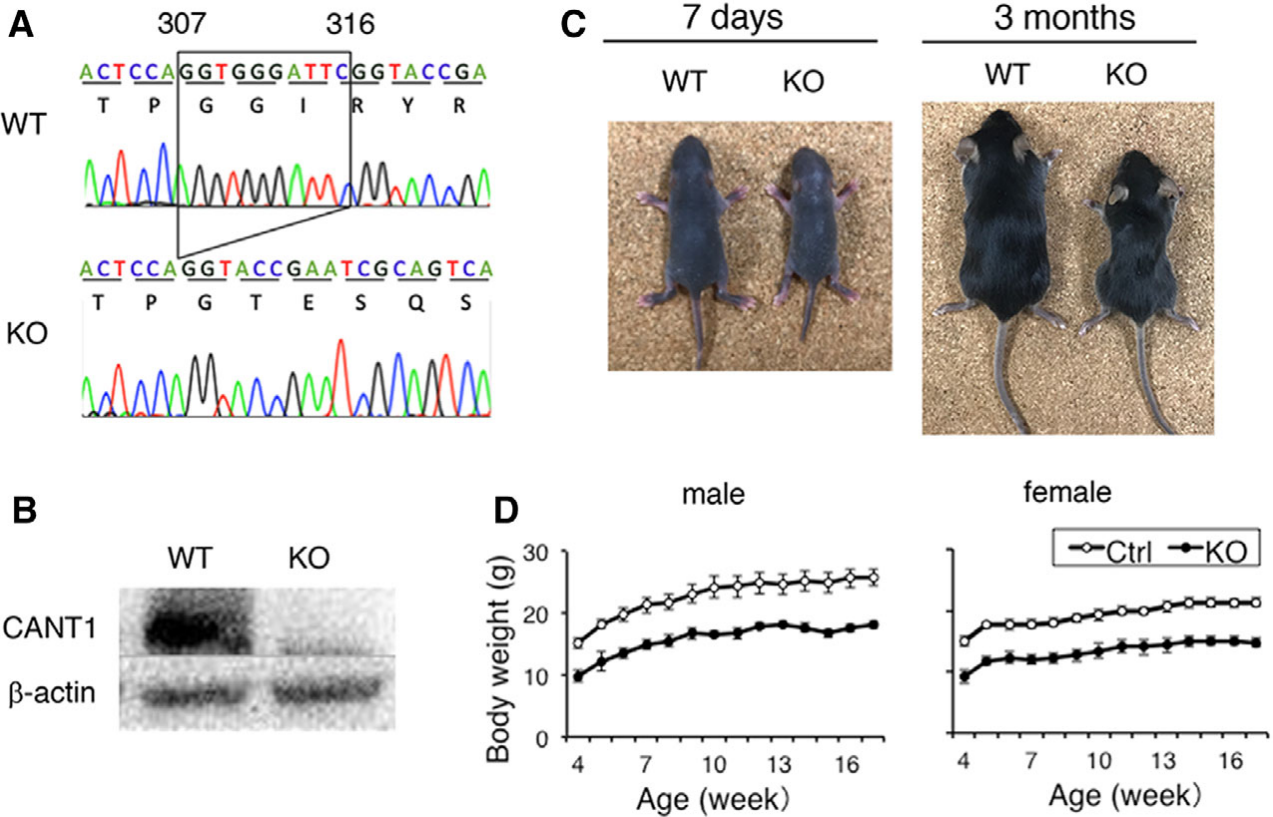
Generation of *Cant1* KO mice by genome editing. (A) Sequence chromatograms of the edited genomic region in exon 3 of the *Cant1* gene. A homozygous 10‐bp deletion (c.307_316del) leading to frameshift occurred in *Cant1* KO mice. (B) Detection of endogenous CANT1 proteins in lung tissue by western blot analysis. (C) Gross appearance at 7 days and 3 months of age. (D) Body weights of *Cant1* KO mice were significantly lower compared with age‐matched control mice. Values represent means ± SD (*n* = 4). *P* values were determined by Student’s *t*‐test. Significant differences (*P* < 0.0001) were observed at all data points. WT, wild‐type mice; KO, knockout; Ctrl, control (WT + *Cant1* heterozygous KO mice).

To generate *Cant1* KO mice, we used the CRISPR/Cas9 system to target exon 3 of the *Cant1* gene. A 10‐bp deletion of the *Cant1* gene coding sequence was expected due to microhomology‐mediated end‐joining repair. Forty‐eight injected zygotes were transferred to pseudo‐pregnant mice, and 15 F0 pups were born. Among them, nine mice exhibited a clear dwarf phenotype. We determined the DNA sequences around the target site of all F0 mice and found that all the dwarf mice had the frameshift deletions at both *Cant1* alleles. The five normal‐sized F0 mice had the frameshift deletion at one *Cant1* allele. These results indicate that the dwarf phenotype was caused by CANT1 deficiency. A stable germline transmission of the edited allele was confirmed in one heterozygous F0 mouse, and *Cant1* KO mice were generated by crossing of the heterozygous breeding pairs. The KO mice were born according to the Mendelian ratio. The homozygous 10‐bp deletion (c. 307_316del) and the deficiency in CANT1 protein were confirmed by DNA sequencing and western blot analysis, respectively (Fig. 1A,B). The dwarf phenotype was recognizable in comparison with WT littermates at 7 days and 3 months of age (Fig. 1C). Body weights of *Cant1* KO mice were significantly lower (*P* < 0.0001) compared with control mice between 4 and 17 weeks of age (Fig. 1D) and likely after that.

### Skeletal and histological defects of *Cant1* KO mice

**Fig. 2:**
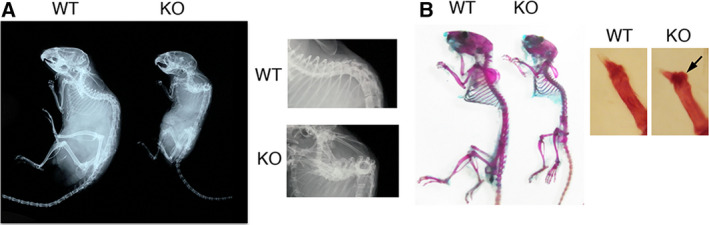
Skeletal defects in *Cant1* KO mice. (A) X‐ray analysis of whole skeletons at 6 months of age. Thoracic kyphosis was present in *Cant1* KO mice. (B) Skeletal specimens of the whole skeleton (left picture) and first digits of the hind limb (right picture) at 3 months of age. A delta phalanx was present in *Cant1* KO mice (arrow). These skeletal defects are similar to those of DD type 1 patients.

**Fig. 3:**
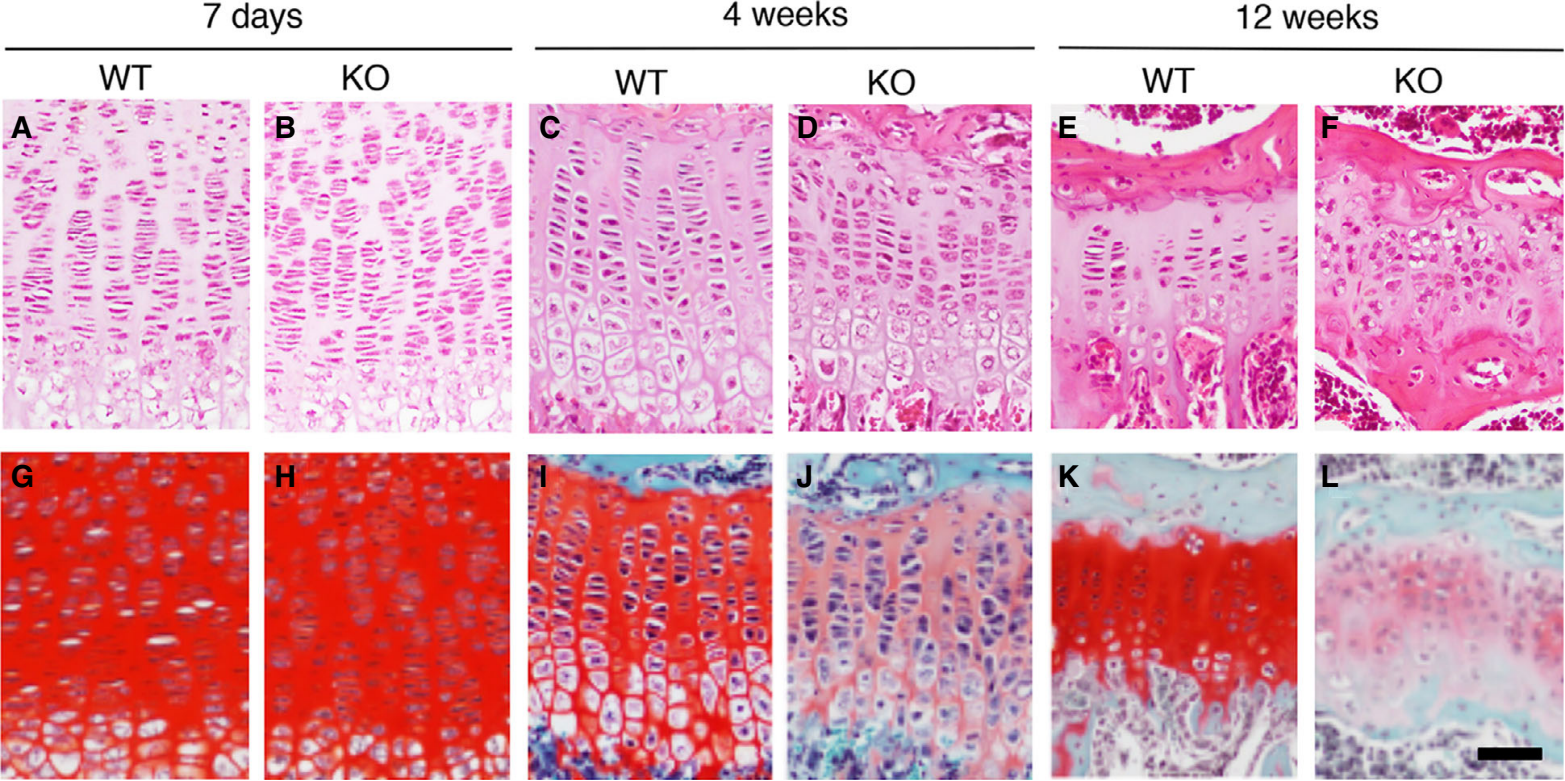
Histological defects in *Cant1* KO mice. Hematoxylin‐ and eosin‐stained sections of growth plate cartilage at 7 days (A, B), 4 weeks (C, D), and 12 weeks (E, F) of age. Safranin O‐stained sections at 7 days (G, H), 4 weeks (I, J), and 12 weeks (K, L) of age. Scale bar = 100 μm.

We investigated skeletal phenotypes by X‐ray analyses and Alizarin red/Alcian blue staining of skeletons. *Cant1* KO mice were smaller compared with WT littermates and demonstrated thoracic kyphosis at 6 months of age (Fig. 2A). There were no clear differences in the double‐staining pattern between WT and mutant mice, but a delta phalanx was observed in the hind limbs of *Cant1* KO mice (Fig. 2B). These skeletal defects, including short stature, thoracic kyphosis, and delta phalanx, are also observed in DD type 1 patients. To understand the pathogenic basis for the skeletal defects, we investigated the histology of the growth plate at 7 days and 4 and 12 weeks of age. The growth plate is composed of three distinct layers of chondrocytes, termed the resting, proliferating, and hypertrophic zones. Proliferating chondrocytes were well spaced by extracellular matrix (ECM) and exhibited typical columnar alignment with a flat shape (Fig. 3A,C,E). The columnar alignment and shape of chondrocytes in the mutants were comparable to those in WT mice at 7 days of age (Fig. 3A,B). At 4 weeks of age, the columnar alignments in the mutants presented a little closer to each other compared with WT mice, due to the decreased ECM space (Fig. 3C,D). At 12 weeks of age, the columnar alignment was completely lost, accompanied by increased cell density due to the decreased ECM space, and the proliferating chondrocytes had lost their typical flat shape and become round (Fig. 3E,F). Safranin O binds sulfated GAGs and stains them intensely red. The intensity of this staining in the mutants was comparable to that of WT mice at 7 days of age (Fig. 3G,H), but was severely reduced at 4 weeks of age (Fig. 3I,J), and even more severely reduced at 12 weeks of age (Fig. 3K,L). These results show that the GAG content in *Cant1* KO cartilage was severely reduced at 4 and 12 weeks of age. Drastic histological changes in the growth plates of *Cant1* KO mice after 12 weeks of age were confirmed by three independent experiments at different ages (12, 15, and 20 weeks of age; data not shown).

### Chondrocyte differentiation was impaired in *Cant1* KO mice

**Fig. 4:**
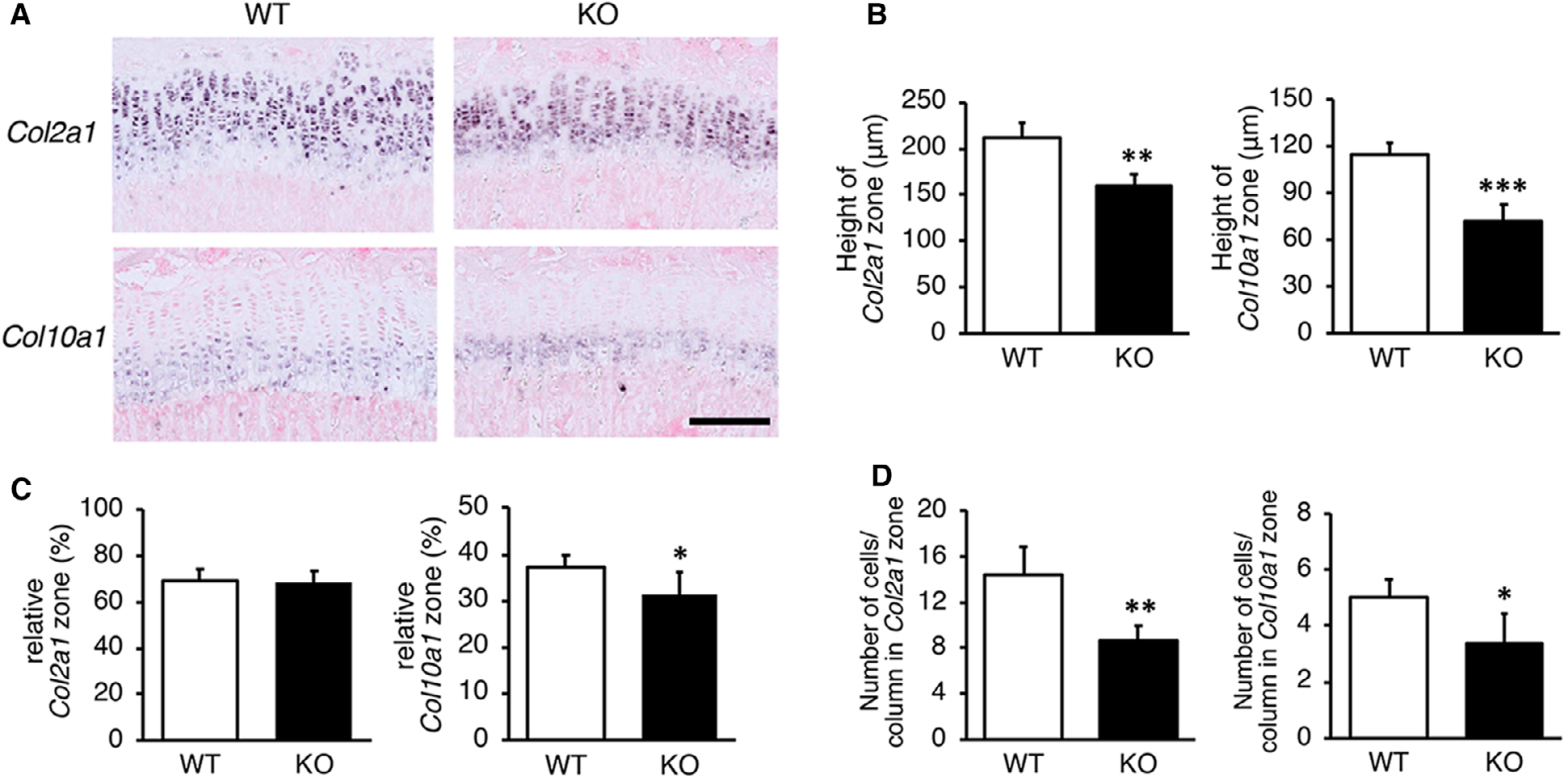
*In situ* hybridization for the detection of chondrocyte marker mRNAs. Sections from the growth plate cartilage at 4 weeks of age were hybridized to *Col2a1* and *Col10a1* probes (A). *Col2a1* is a marker for resting and proliferating chondrocytes, and *Col10a1* is a marker for hypertrophic chondrocytes. Scale bar = 150μm. (B) Heights of *Col2a1*‐ and *Col10a1*‐expressing zones in *Cant1* KO mice were significantly shorter compared with those of WT mice. (C) Relative height of *Col2a1*‐expressing zone normalized by the total height of the growth plate was not significantly different, but that of *Col10a1*‐expressing zone was significantly reduced compared with WT mice. (D) The numbers of chondrocyte per column in *Col2a1*‐ and *Col10a1*‐expressing zones were significantly smaller compared with WT mice. We measured the height and counted the cell number at five different points from a single section. Combined values from two independent sections were compared. Values represent means ± SD (*n* = 10). *P* values were determined by Student’s *t*‐test. *P < 0.05, **P < 0.01, ***P < 0.001.

To investigate the effects of CANT1 deficiency on chondrocyte differentiation, we performed *in situ* hybridization to detect chondrocyte marker mRNAs in sections of growth plates at 4 weeks of age. We used *Col2a1* as a marker for resting and proliferating chondrocytes and *Col10a1* as a marker for hypertrophic chondrocytes. Signals of the two markers were detected in the mutants, as well as in WT mice (Fig. 4A). However, the heights of *Col2a1*‐ and *Col10a1*‐expressing zones in *Cant1* KO mice were significantly reduced (*P* < 0.01 and *P* < 0.001, respectively) compared with WT mice (Fig. 4B; *Col2a1*‐expressing zone, –25.7%; *Col10a1*‐expressing zone, –38.9%). The relative height of *Col2a1*‐expressing zone normalized by the total height of the growth plate in *Cant1* KO mice was not significantly different, but that of *Col10a1*‐expressing zone was significantly reduced (*P* < 0.05) compared with WT mice (Fig. 4C). The numbers of chondrocytes per column in *Col2a1*‐ and *Col10a1*‐expressing zones of the mutants were also significantly reduced (*P* < 0.01 and *P* < 0.05, respectively) compared with WT mice, in parallel with the decreased heights of the respective zones (Fig. 4D), indicating that the decreased heights of the respective zones in the mutants were not mainly due to reduced cell size and/or ECM space. Anti‐PCNA antibody and TUNEL staining of the serial sections showed that there were no differences in the frequencies of either positive cells between WT and *Cant1* KO mice at 4 weeks of age (data not shown), suggesting that chondrocyte proliferation and apoptosis were not affected. These findings clearly suggest that chondrocyte differentiation was impaired in *Cant1* KO mice.

## Discussion

In this study, we generated *Cant1* KO mice using CRISPR/Cas9‐mediated genome editing. The skeletal defects in our *Cant1* KO mice were almost the same as in KO mice previously generated by the ES cell‐based method [19] and are similar to those of DD type 1 patients, including short stature, progressive scoliosis, and delta phalanx. These findings validate the use of *Cant1* KO mice as an animal model for DD type 1.

Reduced GAG content and ECM space, the disorganized columnar alignment of chondrocytes, and the changed shape of proliferating chondrocytes were observed in the growth plates of *Cant1* KO mice at 4 or 12 weeks of age. GAGs are large negatively charged molecules that attract cations and water molecules, resulting in the formation of a hydrated gel. These properties contribute to the maintenance of the optimal compressive stiffness and tissue hydration [21, 22]. Similar histological defects have also been observed in humans and animals that are deficient in GAG synthesis and metabolism [14, 15]. Therefore, the physical properties of ECM conferred by GAGs may be important to maintain the proper columnar alignment and typical flat shape of proliferating chondrocytes.

Paganini et al. showed that, in their *Cant1* KO mice, chondrocyte proliferation was increased and the number of apoptotic chondrocytes was increased in the proliferating zone at 7 days of age, but no differences were detected in either parameter at 14 days of age [19]. Chondrocyte proliferation and apoptosis were not affected in our KO mice at 4 weeks of age. Therefore, the decreased cell numbers in the respective zones in our KO mice should not be mainly due to the defects in chondrocyte proliferation and/or apoptosis but rather due to the impaired chondrocyte differentiation. Higher ratio shortening of *Col10a1*‐expressing zone compared with the *Col2a1*‐expressing zone and significant reduction in the relative height of *Col10a1*‐expressing zone in *Cant1* KO mice indicate that chondrocyte differentiation, especially terminal differentiation to hypertrophic chondrocytes, was impaired in the mutant mice.

Glycosaminoglycan chains have been shown to regulate the distribution and binding ability of several signaling molecules, thereby influencing chondrocyte differentiation [23, 24]. The *Ext1* gene encodes a glycosyltransferase essential for HS chain synthesis. Mice homozygous for the hypomorphic *Ext1* allele (*Ext1^gt^*) exhibited delayed chondrocyte maturation and increased Indian hedgehog (IHH) signaling [25]. On the other hand, conditional *Ext1* KO mice in limb bud mesenchyme exhibited severe chondrodysplasia with dysregulated BMP signaling [26]. The *Chsy1* gene encodes CS synthase 1, which catalyzes the extension of CS and DS chains [13, 14]. In the *Chsy1* KO mice, the expression of *Gdf5,* which encodes a growth factor of the BMP family, is altered during the earliest stages of joint formation, and IHH distribution in the cartilages is also altered in this mutant [27]. A previously reported recessive dwarf mouse mutant (*pug*) possessed a missense mutation in *Xylt1,* a causative gene for DD type 2 [28]. The amount of GAGs was decreased in the cartilage of this mutant, accompanied by the premature maturation of chondrocytes. Broadened expression of Ihh and fibroblast growth factor receptor 3 was observed in the cartilage of this mutant. Based on these findings, it should be determined whether altered signaling leads to impaired chondrocyte differentiation in *Cant1* KO mice.

CANT1 is a nucleotidase that exists as both a membrane‐bound and a soluble secreted form [16, 17, 18]. The membrane‐bound form of CANT1 localizes to the endoplasmic reticulum and Golgi. SLC35D1 is a nucleotide sugar transporter that transports UDP sugars from the cytoplasm into the endoplasmic reticulum [29]. The transported nucleotide sugars are then utilized as substrates for synthesis of CS chains [30, 31]. It has been suggested that the resulting UDP is hydrolyzed to UMP by CANT1, and then, UMP is exchanged via the antiporter system for importing further nucleotide sugars [6, 7, 8, 19]. UDP removal also prevents glycosyltransferase inhibition by negative feedback. On the other hand, the functions of the soluble secreted form of CANT1 remain obscure. CANT1 is a member of the apyrase family, with sequence homology to apyrases cloned from hematophagous arthropods that preferentially hydrolyze ATP and ADP, thereby acting as antihemostatic agents [16, 17, 18]. Therefore, the substrate preference is greatly different between CANT1 and apyrases from the blood‐feeding arthropods, suggesting that they have different functions. CANT1 substrates (i.e., UDP, GDP, and UTP) are involved in extracellular signaling, including calcium (Ca2^+^) release, through activation of pyrimidinergic signaling [32, 33]. The binding of pyrimidinergic nucleotides (UTP/UDP) to P2Y receptors generates inositol 1,4,5‐triphosphate (IP3) through their coupling to phospholipase C. The presence of the soluble secreted form of CANT1 suggests that the enzyme may also modulate cellular responses to extracellular signaling. Generation of gene‐modified mice lacking the signal peptide of CANT1 may resolve this unclarified issue.

## Conflict of interest

The authors declare no conflict of interest.

## Author contributions

KK, HT, and NO performed the main experiments and analyzed the data. KN and TO performed mouse embryo manipulation. KN and EK performed *in situ* hybridization analysis. LG and SI performed genotyping of F0 mice. TF planned the experiments, analyzed data, and wrote the manuscript.
